# Applications of Blockchain in the Medical Field: Narrative Review

**DOI:** 10.2196/28613

**Published:** 2021-10-28

**Authors:** Yi Xie, Jiayao Zhang, Honglin Wang, Pengran Liu, Songxiang Liu, Tongtong Huo, Yu-Yu Duan, Zhe Dong, Lin Lu, Zhewei Ye

**Affiliations:** 1 Department of Orthopedics Surgery, Union Hospital Tongji Medical College Huazhong University of Science and Technology Wuhan China; 2 Laboratory of Intelligent Medicine, Union Hospital Tongji Medical College Huazhong University of Science and Technology Wuhan China; 3 School of Artificial Intelligence and Automation Huazhong University of Science and Technology Wuhan China; 4 Hubei University of Chinese Medicine Wuhan China; 5 Wuhan Academy of Intelligent Medicine Wuhan China

**Keywords:** blockchain, smart health care, health care, health data, review, COVID-19, electronic health records

## Abstract

**Background:**

As a distributed technology, blockchain has attracted increasing attention from stakeholders in the medical industry. Although previous studies have analyzed blockchain applications from the perspectives of technology, business, or patient care, few studies have focused on actual use-case scenarios of blockchain in health care. In particular, the outbreak of COVID-19 has led to some new ideas for the application of blockchain in medical practice.

**Objective:**

This paper aims to provide a systematic review of the current and projected uses of blockchain technology in health care, as well as directions for future research. In addition to the framework structure of blockchain and application scenarios, its integration with other emerging technologies in health care is discussed.

**Methods:**

We searched databases such as PubMed, EMBASE, Scopus, IEEE, and Springer using a combination of terms related to blockchain and health care. Potentially relevant papers were then compared to determine their relevance and reviewed independently for inclusion. Through a literature review, we summarize the key medical scenarios using blockchain technology.

**Results:**

We found a total of 1647 relevant studies, 60 of which were unique studies that were included in this review. These studies report a variety of uses for blockchain and their emphasis differs. According to the different technical characteristics and application scenarios of blockchain, we summarize some medical scenarios closely related to blockchain from the perspective of technical classification. Moreover, potential challenges are mentioned, including the confidentiality of privacy, the efficiency of the system, security issues, and regulatory policy.

**Conclusions:**

Blockchain technology can improve health care services in a decentralized, tamper-proof, transparent, and secure manner. With the development of this technology and its integration with other emerging technologies, blockchain has the potential to offer long-term benefits. Not only can it be a mechanism to secure electronic health records, but blockchain also provides a powerful tool that can empower users to control their own health data, enabling a foolproof health data history and establishing medical responsibility.

## Introduction

### Background

With the development of medical informatization, the amount of available health care data is increasing at an extremely fast rate. The sharing and use of medical information have played an important role in the optimization of medical resource allocation, clinical decision-making assistance, medical quality monitoring, precision medicine, and disease risk assessment and prediction [1-3]. However, such data sharing comes with the risks of data security and privacy concerns, data dictatorship, insufficient autonomy of the subject, increased social unfairness, and others. Moreover, the sudden development of the COVID-19 pandemic has also posed new challenges for personal health data sharing and mining. As another world-changing technology based on cloud computing, the Internet of Things (IoT), and artificial intelligence (AI), blockchain may provide a solution to the above-mentioned problems owing to its unique characteristics such as decentralization, autonomy, credibility, and transparency [4,5]. Therefore, it is necessary to explore the impact of blockchain on the medical industry to further clarify the potential value of the medical application of blockchain technology in the context of medical informatization.

### Objectives

Although previous studies have analyzed blockchain applications from the perspective of technology, business, or patient care, few studies have focused on its actual use-case scenarios in health care. In particular, the outbreak of COVID-19 brought about some ideas for the application of blockchain in medical practice. Therefore, this paper aims to provide a systematic review of the use of blockchain technology in health care. In addition to summarizing the basic principles and framework, this review highlights the different characteristics based on blockchain and the application of blockchain in clinical practice. Furthermore, integration with other technologies is discussed, which provides a reference for future research. Toward this end, we first describe the framework and perform specific technical analyses from a theoretical standpoint. We then summarize the application of blockchain in medical scenarios and sort them into three parts according to the characteristics of blockchain from a practical point of view. Furthermore, we summarize the use cases of blockchain in the fight against the COVID-19 pandemic, including the prevention of infectious diseases, location sharing, and contact tracing, and the supply chain of injectable medicines. Finally, we explore the integration of blockchain with new technology and highlight some of the associated challenges. By depicting a blueprint of interconnected ecosystems in health care, we aim to provide some reflections for engineers and decision-makers in the medical industry.

## Methods

### Design

A systematic review design with narrative methods was used to analyze the existing evidence. More precisely, a review methodology was followed to form a conception of the application of blockchain technology in health care.

### Search Strategy

We performed a comprehensive literature search on May 10, 2021. The following electronic databases were searched with the assistance of an information specialist at a medical library: PubMed, EMBASE, Scopus, IEEE, and Springer. The review was limited to articles published in English between 2016 and 2021 for which abstracts were available. This time frame was chosen based on the dramatic improvement in information technology that occurred during this period. The review was also limited to studies of blockchain technology in the health care domain. The initial search terms used were as follows: (blockchain) OR (distributed ledger technology) OR (smart contract) AND (health care). After reviewing the literature identified through these search terms, we added the search terms “health data,” “clinical,” “biomedical research,” “supply chain,” “drug safety,” and “health monitoring” to capture the relevant studies found in the references of the articles retrieved during the initial search (Table 1).

**Table 1:** Databases and search terms used, and number of references found for each (N=1647).

Database	Search terms	Number of references retrieved
PubMed (MEDLINE)	(((blockchain) OR (distributed ledger technology)) OR (smart contract)) AND (health care)	232
Embase	((blockchain or distributed ledger technology) and health care).af.	185
Scopus	(TITLE-ABS-KEY (block AND chain) AND TITLE-ABS-KEY (health AND care ))	259
IEEE	((“All Metadata”: blockchain or distributed ledger technology) AND “All Metadata”: health care)	295
Springer	blockchain AND in AND health AND care AND “distributed ledger technology” AND (blockchain)	676

### Inclusion and Exclusion Criteria

A total of 1647 search results were screened for relevance using their titles or abstracts, leaving 60 articles that were fully reviewed and summarized. Our inclusion criteria were as follows: (1) application studies that demonstrated the effectiveness of blockchain technology, including data preservation and sharing, medical insurance and supply chain, clinical and biomedical research, drug safety, medical education, electronic prescription anticounterfeiting, wearable devices, and epidemic prevention; (2) English language studies published in scientific journals; (3) studies for which the full text was available; and (4) completed studies. The review was not restricted according to the study location, and any international study written in English was eligible. Our exclusion criteria were papers describing the process of blockchain design, books or book chapters, letters, statistical reviews, dissertations, editorials, and study protocols.

### Study Selection

The research selection included four steps. First, three authors (XY, JZ, and HW) independently screened all titles and abstracts related to the systematic review (N=1647). Second, the abstracts of all related articles were qualified by five authors (PL, SL, TH, YD, and ZD). Third, the full texts of eligible publications were obtained and screened by two authors (XY and LL) according to the inclusion and exclusion criteria. If there were any different opinions in the decisions made, the controversial documents were discussed until a consensus was reached, with the support of ZY. Fourth, the reference list of all included papers and the system overview identified in the original search were checked to identify other publications that met our inclusion criteria.

## Results

### Search Findings

**Figure 1:**
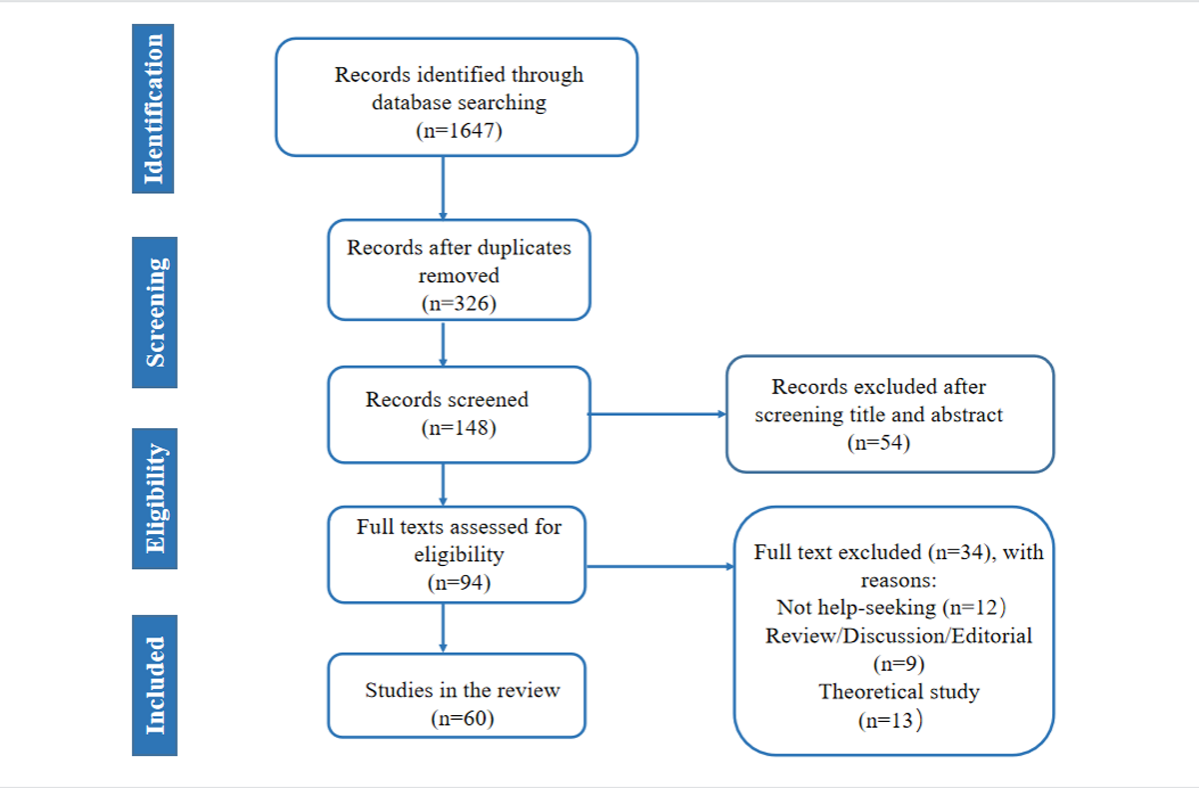
PRISMA (Preferred Reporting Items for Systematic Reviews and Meta-analyses) flow diagram outlining the review process.

The search identified 1647 potentially relevant documents after a review of titles and abstracts, 60 of which satisfied the inclusion criteria after a full-text review (Figure 1).

### Application Principle of Blockchain Technology in Health Care

#### Distributed Ledger and the Characteristics of Blockchain

Although traditional databases can have built-in redundancy, they do not have the advantage of replication on every node [6-8]. In the nodes of blockchain, only cryptography and programs are used to realize point-to-point transactions, complete cooperation, and establish tasks. In this mode, the problems of low efficiency, high cost, and data security caused by centralized systems can be solved [7-9]. Some of the characteristics of blockchain and closely related applications are summarized in Table 2. By maintaining an immutable, tamper-proof, consecutive list of transactional data in a distributed network, blockchain has created several disruptions in incumbent business processes, and provides a promising new distributed framework for amplifying the integration of health care information across a range of stakeholders [1-3,10,11].

**Table 2:** Characteristics of blockchain and related applications.

Characteristics	Description	Related applications
Decentralization	There is no centralized management organization in the whole network, but rather a distributed end-to-end network structure [4-7].	Health data preservation and authorization; the preservation and authorization of health data
Autonomy	Using consensus-based specifications and protocols to enable all nodes to exchange data freely and safely in a detrusted environment [4,5,7,9,10].	Medical insurance; health status monitoring and tracking (wearable devices)
Credibility	Asymmetric cryptography is used to encrypt transaction data, and with the help of workload proof mechanism to ensure that the data are difficult to tamper with in theory [5-7,10].	Controlling of fake drugs; anticounterfeiting of electronic prescriptions; digital ledger of students’ scores
Transparency	All transaction records are open and transparent in the whole network, breaking the information asymmetry [5-10,12].	Supply chain; clinical trials; biomedical research

#### Framework Structure of Blockchain

**Figure 2:**
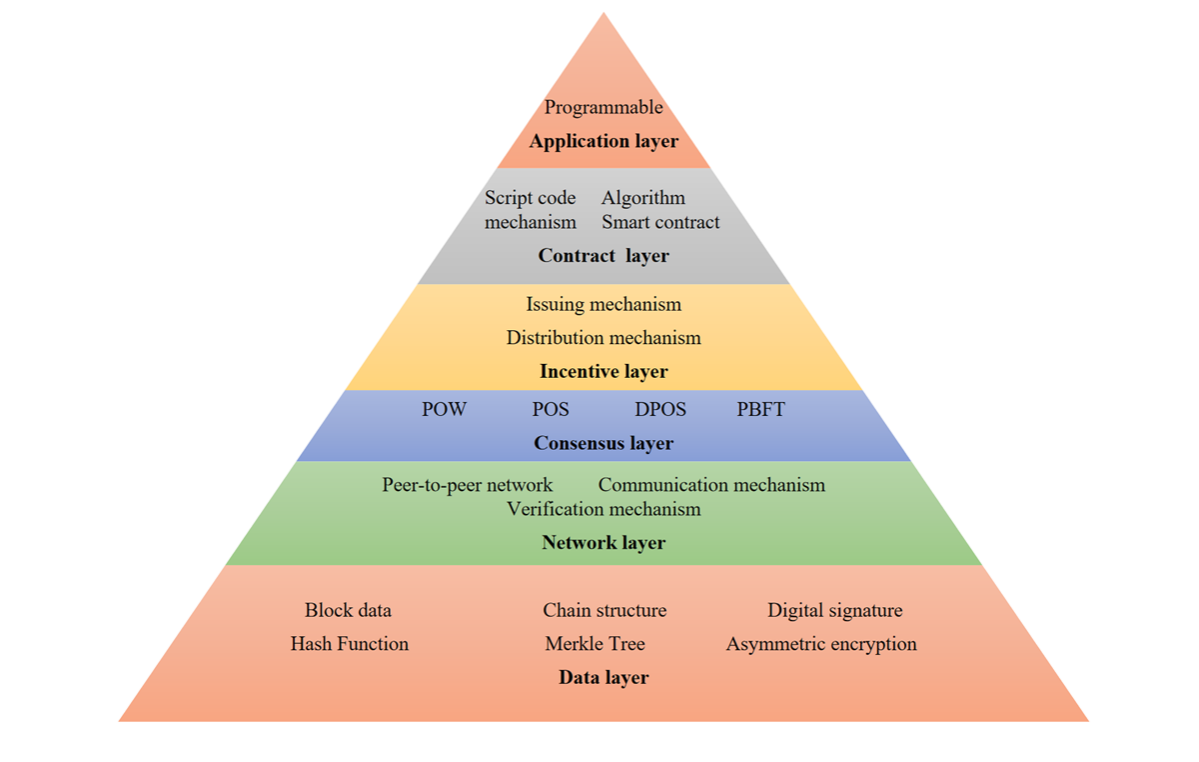
Framework structure and classification of blockchain. DPOS: delegated proof of stake; PBFT: practical Byzantine fault tolerance; POS: proof of stake; POW: proof of work.

The framework and structure of blockchain can be divided into six layers (Figure 2). The application layer carries out the accounting, transferring, and verifying functions on the client side. Then, the contract layer behind the application layer includes the script code, algorithm mechanism, and smart contract, which performs transaction identification. The incentive layer involves the distribution mechanisms. The consensus layer ensures the consistency of the distributed systems and that consensus can be reached even if there are malicious nodes in the network. This layer also avoids the problem of a “double-spend attack’’ and ensures the generation time of blocks. In addition, the network layer is the mechanism for which we use the P2P network to complete communication and confirmation, and the data layer includes a series of encryption and storage technologies [6,7,9,12,13].

#### Classification of Blockchain

Generally speaking, blockchain can be divided into public chains, consortium chains, and private chains, as shown in Table 3 [14]. Hasselgren et al [15] counted the current medical use of various types of blockchains and frameworks, and found that the consortium chain is the most widely used. Currently, ethernet and hyper ledger fabric are the most popular frameworks. Hasavari et al [16] showed that hyper ledger fabric is the most effective frame structure combined with medical treatment at present. One solution to the problem regarding the combination of blockchain and medical treatment is to replace patient care reports with electronic health records (EHRs) so that they can interoperate with other EHRs. Another solution suggests integration of the hyper ledger platform with an interplanetary file system (IPFS), which is a point-to-point method for storing and sharing media in distributed file systems that uses BitTorrent technology. The data themselves are stored on the IPFS, and the hash pointer is stored on the blockchain. MedRec [17] is a decentralized record management system utilizing blockchain for authentication, confidentiality, responsibility, and data sharing. At present, most medical data-sharing and distribution solutions use the allowed blockchain technology and rely on business process integration; that is, customers run code on each node and go through a specific process until they store the data in the ledger [18].

**Table 3:** Classification of blockchain [14].

Property	Public blockchain	Consortium blockchain	Private blockchain
Consensus determination	All miners	Selected set of nodes	One organization
Read permission	Public	Could be public or restricted	Could be public or restricted
Immutability	Nearly impossible to tamper	Could be tampered	Could be tampered
Efficiency	Low	High	High
Consensus process	Permissionless	Permissioned	Permissioned

### Application Scenarios in Health Care

#### Principal Scenarios

Blockchain technology can use cryptography to program and operate smart contracts composed of data encryption and automated script codes, and also provides distributed infrastructure and economic incentives [4,5,10,11,13,14,17-19]. Based on previous studies, we summarize three main blockchain-based medical scenarios (Table 4) and discuss the integration of blockchain and emerging technologies in the future.

**Table 4:** Medical application scenarios of blockchain and the details of related studies (N=60).

Applications according to technical characteristics		Number of studies		References
**Preservation and authorization of personalized health data**				
	Preservation of health data	10	Kim et al [6], Shi et al [20], Zhou et al [21], Dubovitskaya et al [22], Jones et al [23], Chen et al [24], Hylock et al [25], Lo et al [26], Xiao et al [27], Yue et al [28]	
	Medical data sharing	8	Xia et al [29], Patel et al [30], Fan et al [31], Yazdinejad et al [32], Zhu et al [33], Dubovitskaya et al [34], Khurshid et al [35], Cheng et al [36]	
	Medical insurance	1	Zhou et al [37]	
	Health status monitoring and tracking	3	Brogan et al [38], Griggs et al [39], Ichikawa et al [40]	
**Promoting the management of social and public health**				
	Administration of medicine	8	Mao et al [41], Fernández et al [42], Sylim et al [43], Lohmer et al [44], Hoy et al [45], Vruddhula et al [46], Tseng et al [47], Mackey et al [48]	
	Anticounterfeiting of electronic prescriptions	2	Aldughayfiq et al [49], Li et al [50]	
	Prevention and control of pandemic	10	Raghavendra et al [51], Nandi et al [52], Marbouh et al [53], Mashamba et al [54], Bansal et al [55], Khurshid et al [56], Abdel-Basset et al [57], Resiere et al [58], Chang et al [59], Garg et al [60]	
**Empowering the credibility of medical education and research**				
	Medical education	3	Verde et al [61], Funk et al [62], Durant et al [63]	
	Clinical trials	7	Benchoufi et al [64], Wong et al [65], Omar et al [66], Hirano et al [67], Zhuang et al [68], Nugent et al [69], Wan et al [70]	
	Biomedical research	8	Jin et al [71], Kuo et al [72-74], Ozercan et al [75], Johnson et al [76], Chen et al [77], Mamoshina et al [78]	

#### Personalized Health Data Preservation and Authorization

##### Overview

With the accumulation of a large amount of individual health information, a reliable storing and sharing approach is needed to ensure the safety of patients’ private information. The existing medical data management systems are generally based on a set of centralized servers, which build a large site system or centralized relational database system. Blockchain is an open distributed ledger based on peer-to-peer networks and consensus algorithms with natural advantages in solving these problems.

##### Preservation of Electronic Medical Records

With the increased specialization of health care services and high levels of patient mobility, accessing health care services across multiple hospitals or clinics has become very common for diagnosis and treatment, particularly for patients with chronic diseases. Based on blockchain, Dubovitskaya et al [22] developed ACTION-EHR, an EHR data management system for the radiation treatment of cancer. The synchronous nodes in the blockchain network can immediately find data changes and prevent malicious tampering with the data. Similarly, HealthChain, a novel patient-centered blockchain framework designed by Hylock and Zeng [25], offers patients and providers access to consistent and comprehensive medical records. To integrate patient referral data from the National Health Insurance Administration national medical referral system, Lo et al [26] developed a blockchain-enabled framework for acquiring electronic medical record (EMR) and EHR data of patients in hospitals and community-based clinics. The framework assists in the establishment of an alliance-based medical referral service to promote trusting relationships and transaction security among patients, family doctors, and specialists. In addition, Yue et al [28] proposed the Health Care Data Gateway, a medical data network that not only enables a patient to control their own data easily but also allows untrusted third parties to process health data securely. By expounding the working principle and process of HealthChain in detail, Xiao et al [27] verified the feasibility of using blockchain in EMRs. As a major direction of telemedicine, the blockchain-based storing system of health data will play a vital role in protecting the privacy of patients and ensuring credibility.

##### Exchange of Patients’ Medical Data

Traditional medical records are stored in the central database of various hospitals, which leads to the phenomenon of an “isolated island of information” in the medical field [1-3,29,31,32,35,36,79-83]. Blockchain allows patients to have access to their medical information while authorizing them to grant access of their EHRs to third parties as they see fit. Health care providers have easy access to all patients’ medical data, regardless of when and where health care services are provided. Guardtime [79], a Netherlands-based data security firm, partnered with the government of Estonia to create a blockchain-based framework to validate patient identities when sharing their health records. Xia et al [29] introduced MeDShare, a system that addresses the trust issue of medical data sharing among medical big data custodians in an untrusted network environment. For example, it may be urgently necessary to know the radiation dose received during treatment to avoid possible harmful consequences for the patient. In a clinical imaging department, a smart framework for cross-domain radiological image sharing and patient-defined access permissions was developed. Zhu et al [33] proposed a cloud resource–sharing model and explored the cloud service of breast tumor diagnosis based on a consensus-oriented blockchain to protect the privacy of cancer patients. In clinical work, timely and accurate sharing of private data related to personal health is necessary for patient treatment, and the significance of blockchain in this respect should be realized by more doctors and decision-makers.

##### Simplifying the Process of Medical Insurance

**Figure 3:**
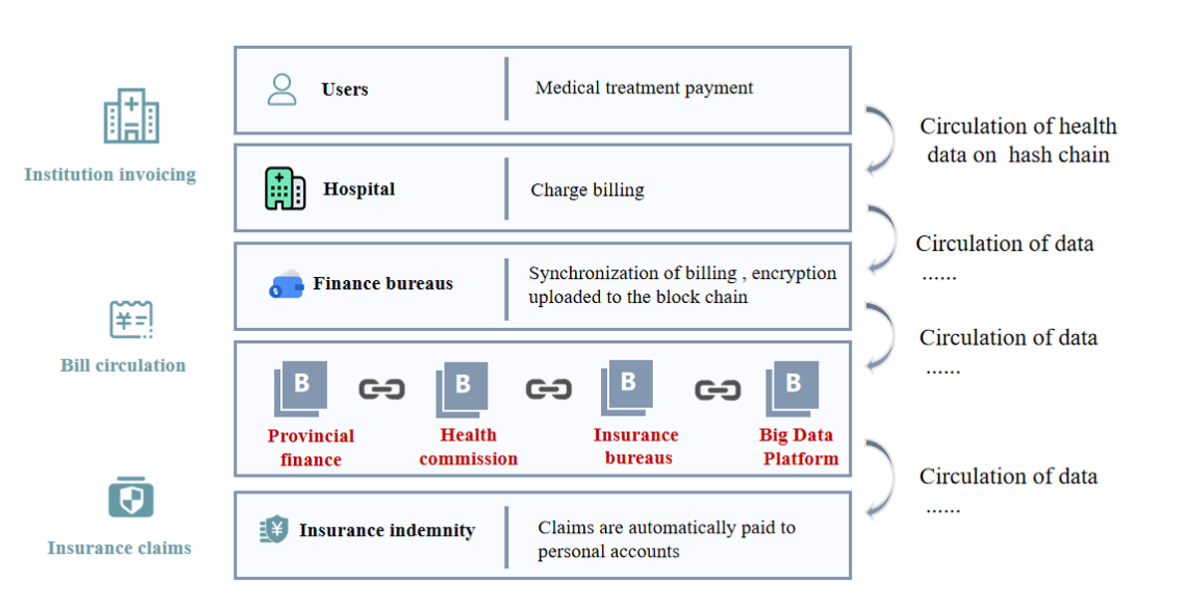
Medical insurance on blockchain.

In the field of medical insurance, the insurance process involves patients, medical institutions, and insurance service providers; however, the speed of information exchange among them is slow. Based on blockchain, the process can be simplified. Zhou et al [37] proposed a blockchain-based medical insurance storage system named MIStore, which was deployed on the Ethereum blockchain and provided a platform between insurance companies and hospitals. The system improved the efficiency of the information storage process so that insurance companies can quickly settle claims and preauthorize payments to patients. Based on blockchain, the real-time circulation and sharing of bills can be realized, which can ensure the payment of claims and reduce user advances. The technical framework of medical insurance based on blockchain is depicted in Figure 3.

##### Monitoring Health Status Based on Personal Wearable Devices

Chronic disease is the dominant cause of morbidity and mortality worldwide. The cost of treating traditional chronic diseases such as cardiovascular disease was US $555 billion in the United States in 2014 and is estimated to rise to US $1.1 trillion by 2035. With the extensive development of wearable devices, smartphones, clouds, and wireless systems, the integration of these devices can be applied to tracking the health status of patients with chronic diseases. By using blockchain-based transmission and storage mechanisms, health information can be uploaded to the cloud more accurately and in a more timely manner [84-86]. Brogan et al [38] demonstrated that it was feasible to use a distributed ledger to receive authenticated activity data from a wearable device. Griggs et al [39] proposed the utilization of blockchain-based smart contracts to evaluate information collected by health care devices and log transactions in a wireless body area network. A quick-response system based on the blockchain can detect emergencies such as asthma attacks and alert the closest emergency services in real time to provide immediate relief. Continuous monitoring can not only detect adverse health events early and reduce the risk of such events but can also improve the monitoring of medication compliance and reduce unnecessary treatments [40,87]. Based on blockchain, wearable devices can potentially reduce patients’ needs for more complex interventions, which in turn reduce the number of emergency department visits and hospitalizations, caregiver burden, and health care costs.

#### Promoting the Management of Social and Public Health

##### Overview

The management of social and public health is involved in many aspects, including disease prevention and control, management of drugs, authentication of health records, and medical insurance. Due to the lack of information exchange, the process often becomes inefficient. Based on the characteristics of blockchain such as traceability and immutability, we can apply it to simplify the process of insurance, administration of medicine, anticounterfeiting electronic prescriptions, and controlling pandemic issuance.

##### Administration of Medicine

The Center for the Public Interest in the United States estimates that global sales of counterfeit drugs are likely to exceed US $75 billion this year, representing an increase of 90% within the past 5 years. According to the World Health Organization, 10% of the world’s medicines are counterfeit drugs, 30% of which are found in developing countries. Owing to their substandard dose and purity, these counterfeit drugs pose a great threat to people’s physical and mental health [41,88,89]. In the supply chain, supply chain management systems have played important roles and have great importance for an enormous number of industries and organizations. With the development of mobile technologies, the supply chain of drugs is considered relatively mature and easy to adopt; the existing solution is to track the logistics information and check the authenticity of drugs through quick response codes, radio frequency identification, or SMS text messaging; however, these methods do not prevent counterfeit drugs from entering the logistics chain. The combination of supply chain and blockchain technologies can revolutionize the medical field, bringing about the benefits of vast objects’ connectivity, and features to process and record a large amount of medical information with more efficiency, privacy, and security [89].

Due to the particularity of drug production, the legitimacy and authenticity of drugs can be effectively guaranteed by using blockchain anticounterfeiting technology [45,46]. Using blockchain, a supply chain can maintain the privacy and security of information and provide great immutability for transactions. For example, Mao et al [41] provided a blockchain-based credit evaluation system to strengthen the effectiveness of supervision and management in the food and drug supply chain. Based on the concept of Industry 4.0, Fernández-Caramés et al [42] presented the design of an unmanned aerial vehicle and blockchain-based supply chain system for traceability applications. To develop a supply regulatory system for drugs, Sylim et al [43] developed a distributed application based on smart contracts. Blockchain has interesting features of validation and smart contracts, which are very useful for medical supply chains to manage millions of transactions in more authentic ways [44]. In the pharmaceutical industry, Chronicled Inc launched a prototype technology combining near field communication–embedded adhesive seals that were registered and verified on the blockchain. Vruddhula [46] launched a project called Oggic to prevent the entry of counterfeit drugs into the supply chain. Tseng et al [47] chose the Gcoin blockchain to address the anticounterfeiting problem in the pharmaceutical world. Each transaction would be recorded on the blockchain, making it tamper-proof, decentralized, time-stamped, and highly secure. Mackey et al [48] proposed a framework for improving the claims process by using blockchain, which makes the adjudication process more patient-centric and prevents drug fraud and abuse.

The primary function of blockchain in the circulation of drugs can be summarized as follows: (1) track and trace pharmaceutical raw materials and finished products in an immutable digital ledger, (2) provide greater transparency of fake drugs by allowing participants to verify their authenticity, (3) integrate anticounterfeit devices into the IoT and provide better authentication, and (4) serve as an underlying technology to enhance information exchange across different actors in the drug supply chain [47,48].

##### Anticounterfeiting of Electronic Prescriptions

**Figure 4:**
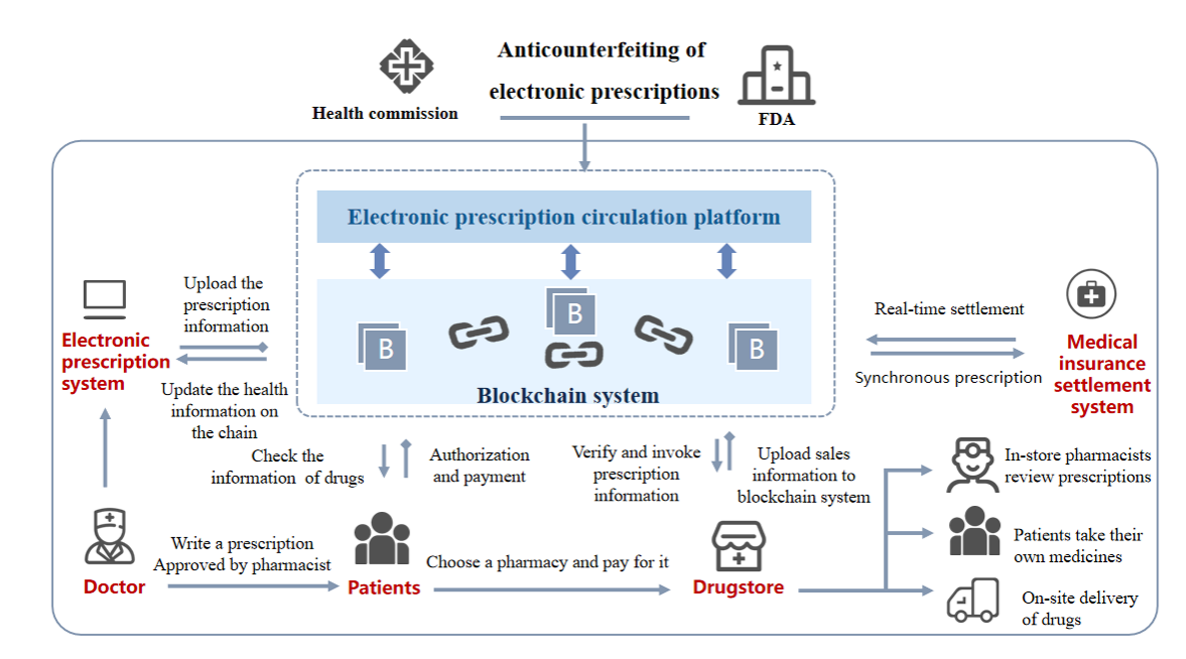
Structure for anticounterfeiting of electronic prescriptions. FDA: US Food and Drug Administration.

With the continuous development of medical treatment on the internet, the establishment of online consultation platforms has facilitated consultation needs. Currently, individuals can conduct medical consultations through online platforms, purchase drugs from certified online pharmacies, and provide services such as drug delivery in the same city [49]. However, fake symptoms and irregular electronic prescriptions continue to emerge, which lead to the behavior of relying on fake prescriptions to buy drugs. Based on the decentralized and traceable blockchain system, a blockchain online consultation platform can be established to ensure the storage of large sample data and the sharing of health information [2,3,5,11]. The framework is shown in Figure 4. Such a project mainly realizes the functions of hospital management, physician management, user management, online consultation, and prescriptions through a doctor-patient online consultation platform, and achieves information credibility with the help of blockchain digital authentication, integral management, and other technologies. In this framework, users can be divided into doctors, ordinary users (patients), and administrators. Doctors need to pass the qualification examination to achieve the functions of prescribing medicine and diagnosing diseases, while patients need to register their personal information and submit a description of their illness to obtain a prescription. The consultation information is uploaded and recorded in personal medical records to prevent patients from fabricating false health information [50]. According to the results of physical examination records, medical diagnosis, and prescription opinions uploaded, the above-mentioned process can ultimately be realized in this blockchain-based tamper-proof system.

##### Pandemic Prevention and Control (Case of COVID-19)

COVID-19 spread rapidly around the world during 2020, which has attracted substantial attention on methods to prevent and control epidemics. Scientists are struggling to find a model to fight COVID-19. Blockchain technology has several potential use cases that can help tackle the current pandemic crisis. Blockchain can be used to simplify the clinical trial processes for vaccines and drugs, raise public awareness, transparently track donations and fundraising activities, and act as a reliable data tracker. Compared with traditional supply information management, blockchain smart contracts can guarantee the rights and obligations between nodes and the judgment of contract execution [51-53,56,58,59]. A joint Walmart-IBM project demonstrated how tracking the sources of contamination in green vegetables, a task that previously took months, could be achieved within seconds using blockchain [53]. Thus, many experts have made meaningful attempts in the control of epidemics and pandemics. Mashamba et al [54] proposed a blockchain-based and AI-coupled self-testing and tracking system for infectious diseases. Based on blockchain, International Classification of Diseases codes (current and previous versions) can be used to map the causes of death and observe disease trends and pattern changes across geographical locations over time. Bansal et al [55] suggested the use of a blockchain-based system to mitigate the falsification of test reports and encourage people to contact individuals with immunity-based licenses. Blockchain also enables information to be collected from individuals without identifying them by using a system of public and private keys. The DeepTrace system based on the blockchain can provide anonymized personal identification while allowing regulators and health care providers to contact people who are at risk of being potentially infected [56]. In addition, Abdel-Basset et al [57] proposed a framework integrating different disruptive technologies and blockchain to provide governance with an integrated vision toward managing the COVID-19 pandemic. Although epidemics and pandemics have seriously affected our lives, they also promoted the advancement of scientific technologies in the process of combating viruses and enhanced our capabilities in facing such emergencies [58-60].

#### Empowering the Credibility of Medical Education and Research

##### Overview

Medical education and research are promising areas where the introduction of blockchain may bring about benefits. Based on a blockchain system, reports and procedures at different stages of the study, as well as test results, and congress and course attendance in medical education could be easily archived and serve as digital proof of acquired competencies performed [62,63]. Since real clinical and experimental data are inseparable from high-quality medical research, the source and reliability of data are essential for researchers [64-66,68,90]. Based on the immutability and transparency of blockchain, the uploading of experimental data records can accelerate clinical data sharing and prevent academic misconduct [69].

##### Medical Education

Medical education is constantly changing and must adapt to address advances in biomedical sciences, improvements in learning theory, new regulatory policy, technological innovation, and efforts to have health care professionals perform at the highest level of competency. Blockchain technology in medical education has the potential to help solve many of the challenges currently faced by academic administrators, faculty, learners, and institutions. Since medical education is a lifelong learning process, a blockchain framework and measurable public exchanges between learners and teachers allow for the transmission of content, feedback about instructional designs, evaluation of learners, competency assessment, and certification.

A blockchain-based structure for the recording, crediting, and appraisal of educational deliverables could be a robust way for educators to track the value that their academic and system achievements create. In medical schools, blockchain can be used to store and track students’ scores and abilities acquired through a range of different clinical environments. Doctors can then decide whether they are willing to share such information so that verified certificates and diplomas can be issued more easily and the process can be more cost-effective and tamper-proof [61,62]. Verde et al [61] proposed that the introduction of blockchain in academic radiology settings can be valuable for monitoring resident progress over the years. Massachusetts Institute of Technology launched a pilot program in 2017 involving the issuance of digital diplomas to students’ smartphones via an app called Blockcerts Wallet, which is based on blockchain [63]. Blockchain could optimize the use of administrative resources by reducing the bureaucratic workload, with the added benefit of increased transparency, as records stored via blockchain can be automatically verified. Objectively speaking, blockchain implementation in the educational system could be in the assessment of faculty member competencies and academic performance in a secure and unalterable fashion.

##### Clinical Trials

The integrity of data in a clinical trial is essential; however, the current data management process is too complex and highly labor-intensive. By applying blockchain technology to medical research, the data can be time-stamped and transparent [64,65]. Wong et al [65] illustrated that a blockchain-based file and data structure could be used to reliably safeguard data in a clinical trial network. Omar et al [66] proposed a blockchain-based framework for computed tomography (CT) data management in clinical trials. Hirano et al [67] performed a project to demonstrate data management under a regulatory sandbox and tested the system through a clinical trial for breast cancer. Cichosz et al [91] explored the operational concept of the use of blockchain to improve data management and analyze diabetes in clinical observations. Nugent et al [69] showed that smart contracts can act as trusted administrators, which can improve the transparency of data reporting in clinical trials. In addition, Engel et al [92] proposed that blockchain can play an essential role in improving surgical outcome research and trial design. Even before clinical trials begin, all plans, agreements, scenarios, and possible results can be stored on blockchain [70]. This approach can transform our thinking about trial design and produce truly veriﬁable and immutable data, which in turn can lead to better data reproducibility.

##### Biomedical Research

Biomedical data sharing has always been a cornerstone of scientific development. In the open world of science, it is inevitable to share, access, analyze, and learn from different sources of data for a meaningful result [73,74,77,93]. Blockchain can not only help in clinical trials but can also accelerate biomedical research and reduce reporting selectivity and fabrication, which are widespread problems in today’s science fields. This can be achieved by integrating the consensus model of blockchain into current solutions to decentralized data storage and analysis. Jin et al [71] introduced LifeCODE.ai, a blockchain-based genomics big data platform, which aims to provide relatively safe and trustworthy data storage for genomic stakeholders. This is a decentralized approach in which each owner has complete control over their data, including where it is stored, who can access it, and when it is updated. This approach may be the best way of sharing scientific data.

The majority of the recent approaches to personalized medicine in oncology and other diseases have relied on various data types, including multiple types of genomic, transcriptomic, microRNA, proteomic, antigen, imaging, physiological, and other data. Research institutions can use DNA data stored in the blockchain to perform advanced searches to find topics of interest for potential genomic research. However, biomedical data are often personal, private, and sensitive, and should thus be treated carefully. There are currently a few similar proposals to help protect the data for academia. The first is the Cancer Gene Trust being developed by the Global Alliance for Genomics and Health Consortium, and the second is the CrypDist project. Both projects have similar properties, where summary data such as somatic cancer variation data are kept and distributed in a blockchain system [75]. In addition, Johnson et al [76] described a decentralized app to build a secure biomedical data-sharing system in biomedical and health care communities. This unprecedented progress has brought us into an era of genomic data–driven medicine and drug development, and blockchain technology will bring us into an era of genomics in an all-around way [77,78,93].

#### Integration with Emerging Technology in Health Care

Currently, the combination of the unsustainable cost of care, an aging population, the need for improved access to care, and the growth of precision medicine has ignited the ideal platform for disruptive innovation through blockchain and digital health [1-3,77,78,94-97]. Based on the distributed ledger, blockchain can be integrated with AI, cloud computing, big data, and the IoT, gaining more application scenarios and aiding the development of the health industry [78,94]. We summarize the practical usage of blockchain in Table 5.

**Table 5:** The integration of blockchain and emerging technology.

Emerging technology	Integrating with blockchain	The role of blockchain	Reference
AI^a^	Disease diagnosis and prediction, medical image–assisted reading, intelligent devices, new drug research, health management, and gene sequencing	Blockchain forms a natural foundation to standardize health data structures for AI training, clinical trials, and regulatory purposes. Blockchain-based AI could thereby accelerate the definition of phenotype-specific outcomes of orphan diseases, improve the representation of racial minorities, and reduce sex-specific inequalities	[94,95,98,99]
Cloud computing	Supporting communication and sharing data among stakeholders in health care	Based on the blockchain and cloud computing, the prediction model of disease evolution will be constructed, which plays a role in the prevention and control of major infectious diseases and tumors involving personal information	[82,83,100]
Big data	Offering a huge amount of data in real time to reduce health risks and optimize the outcome	Big data are stored and shared through a blockchain, thus avoiding the defect that centralized storage is easy to be lost and attacked	[77]
Wearable devices	Collecting personal data in an intimate and timely manner, the source of health data for telemedicine	Blockchain allows continuous individual monitoring, and requires robust, rapid, real-time analysis of physiological signals to avoid the storage of large amounts of data in a centralized system	[96]
Internet of Things	Supply chain and tracing of drugs, apparatuses, and wearable devices	Tracking the source and use of drugs and medical devices in the Internet of Things through blockchain technology to ensure authenticity and reliability	[88,100]
5G	Improving the speed of medical information sharing	The distributed processing of medical data in the 5G environment is carried out by blockchain technology, and access and use can only be achieved by obtaining multiparty permissions	[97]

^a^AI: artificial intelligence.

As shown in Table 5, AI can play a more sophisticated role in patient care through diagnostics, treatment, prevention, and predictive modeling [95,98,99]. For instance, in the cardiovascular arena, convolutional neural networks were recently trained using electrocardiograms (ECGs) from one specific wearable monitor in patients and provided cardiologist-level diagnostic accuracy for arrhythmias. An example is the smartphone app KardiaBand from AliveCor [94] based on machine learning for the identification of atrial fibrillation episodes from ECG data. Pilozzi et al [95] used natural language processing and blockchain-based storage systems to alleviate Alzheimer disease stigma and fears among patients. To diagnose early cancer from CT images, Kumar et al [98] proposed a novel multimodel method combining deep learning and blockchain technology, which showed a perfect effect in practice.

Additionally, cloud computing is a new technique that provides different services by minimizing cost and infrastructure, and can be used with the blockchain system to support communication and sharing data among stakeholders in health care [82]. Liang et al [83] presented ProvChain, a blockchain-based data provenance architecture, to provide assurance of data operations in a cloud computing and storage application, while simultaneously enhancing privacy and availability.

**Figure 5:**
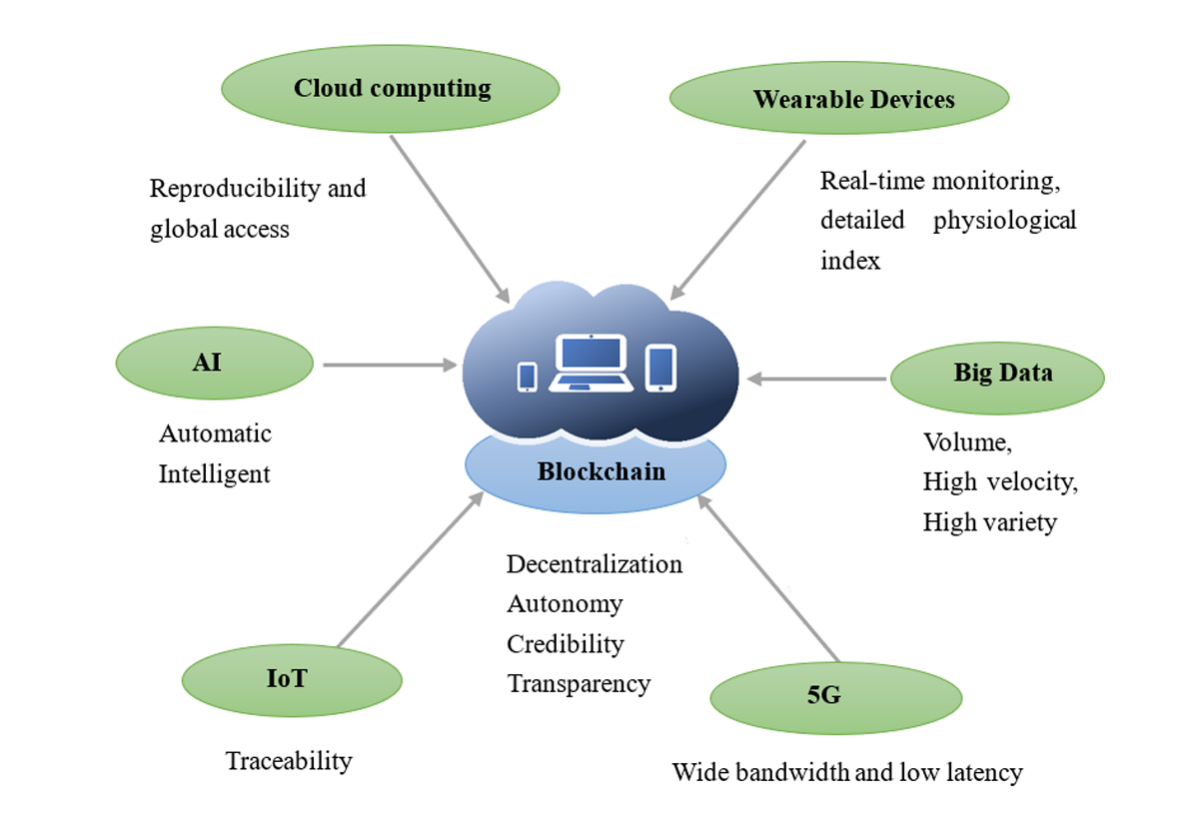
Advantage of integration based on blockchain and emerging technologies. AI: artificial intelligence; IoT: Internet of Things.

Furthermore, big data can be generated from different sources such as wearable devices, EHRs, magnetic resonance imaging, and CT imaging. By integrating information about multiple features of diseases, big data offer an enormous amount of data in real time to reduce health risks and optimize health outcomes [77,82,83,96]. As we discussed in a previous study [85], wearable devices can be used to perceive, record, analyze, regulate, and intervene to maintain health, and can even be used to treat diseases with the support of various technologies for identification, sensing, connection, cloud services, and storage [96,100]. IoT can then play an essential role in the supply chain and tracing of drugs, apparatuses, and data from wearable devices. Based on blockchain and IoT, Fernández-Caramés et al [100] described the design and implementation of a system that enhances continuous glucose monitoring by adding IoT capabilities to allow for monitoring patients remotely and warning them about potentially dangerous situations. To motivate users to add new data to the system, an incentive system based on a digital cryptocurrency can be devised to reward the users that contribute to the system by providing their own data. This is a meaningful attempt to control chronic diseases. Furthermore, in the 5G environment, the blockchain-based cloud system is expected to take full charge of data transfer, storage, and processing. By combining all of these technologies, we can build a healthy database for developing a complete medical ecosystem (Figure 5).

## Discussion

### Principal Findings

The results of this narrative literature review suggest that blockchain is an innovative technology with great potential in health care. Prior reviews included conceptual papers, industry reports, and empirical research that primarily focused on technology, business, or patient care. In this paper, we demonstrate the use of blockchain in managing medical data, confirming the traceability of the supply chain, and in anticounterfeiting electronic prescriptions and clinical or biomedical research. We also demonstrate the important role that blockchain has played during the COVID-19 outbreak, which provides a reference for the prevention of major infectious diseases in the future. The integration and application of new technologies and blockchain were explored, attempting to describe a blueprint of the interconnected ecosystem in health care.

From a practical point of view, the application of EMR exchange appears to be relatively mature. For example, in 10 studies, there were some related systems such as ACTION-EHR [22], HealthChain [25], Healthcare Data Gateway [28], Guardtime [79], and MeDShare [29], which are based on blockchain and used for preservation and exchange of health data. In five studies [42-45,47], the supply chain of medicine appeared to be another important area where blockchain may function. It would make sense to certify medical devices and monitor health status in combination with wearable devices. Moreover, the usage of tracing medicine will prevent the spread of fake drugs. In addition, based on a highly transparent scientific system in clinical and biomedical research, we can enhance the efficiency and confirm the integrity of the study. The situation for the prevention and control of COVID-19 remains grim, and many scholars (eg, [51-60]) have proposed using blockchain technology for preventing and controlling the epidemic, tracking asymptomatic infected individuals, and distinguishing former infected individuals. This decentralized technology ensures the patients’ privacy and protects their rights and interests. The progress of electronic information technology has brought about the development of interdisciplinary tools with a benefit to the medical industry [1,3,64,95,97-99,101-103]. Undeniably, blockchain is booming under the influence of disruptive technologies. These current new technologies are constantly merging with blockchain to innovate medical models and systems. We listed the six typical integrations (AI, cloud computing, big data, wearable devices, IoT, and 5G) with blockchain and wish to provide readers with some inspiration.

However, some concerns were also identified when applying blockchain to health care. The first was the interoperability issue. When encountering a problem requiring cooperation, it is necessary for blockchain-based service providers and users to connect seamlessly; however, the standards among different institutions are not unified. There still exist great differences in the supervision modes of blockchain across countries. In the European Union, individual countries may be willing to use blockchain technology for public plans, but it is not clear how blockchain projects meet the EU General Data Protection Regulation privacy standards [1-4,96,97,104]. The general standard of blockchain will accelerate the industry to reach an agreement on blockchain and contribute to the formation of a large-scale ecosystem of social blockchain [101-103,105-107]. The second is efficiency issues. With the amount of data growing exponentially, the blockchain database has higher requirements for network speed, and the efficiency of data dissemination and real-time acquisition of data will be affected. More reasonable frameworks need to be designed to avoid blockchain efficiency problems across sectors. Moreover, security issues must be taken into consideration. It is not clear whether blockchain is truly the solution for all issues regarding highly sensitive data. The decentralized networks may be subject to 51% of attacks when there are fewer honest nodes than malicious nodes in the network, and the entire network will be taken over by malicious attackers. The last but not least concern is related to regulatory policies, which are important factors for the stable development of blockchain. Decentralization is one of the advantages of blockchain; however, after diluting government regulations, blockchain may have an impact on the existing electronic health system of a country [1-3,97,101,102,104-106]. Therefore, related policies need to be introduced systematically as soon as possible.

### Limitations

The results of this review must be interpreted with caution owing to multiple limitations. First, the findings of this scoping review are mainly intended for health care entities and are not as applicable to other domains such as business and marketing. Second, for practical reasons, the search strategy was restricted to studies reported in the English language, which could have overlooked other benefits and threats reported in other studies in languages other than English. Third, owing to the broad variety of application scenarios of blockchain in the medical field, we cannot demonstrate every aspect of principle and framework in detail.

### Conclusions

With the continuous improvement and development of new technologies, blockchain may become increasingly closely integrated with the contemporary development of the financial sector and health industry. Since each country and region has different attitudes toward this technology, we need to conduct in-depth exploration and research on the blockchain according to individual situations. In medical applications, many startup companies are actively exploring and promoting the development of blockchain in the fields of posttransaction settlement, smart contracts, supply chains, and identity authentication. From the theoretical perspective, blockchain-based theoretical foundations have been established for enhancing trust in an intelligent medicine environment. In the future, the issuance of digital currency will change the traditional economic transaction mode, and the introduction of blockchain will reshape the value exchange system, increase trust and privacy, and efficiently complete economic transactions and medical records [108]. Although countries and regions worldwide have diverse attitudes toward the blockchain, along with skeptical attitudes, these will not affect the research and further development of this technology. Throughout this work, we have also highlighted the principles and major challenges concerning distributed ledger technology. The great value of blockchain-based health care systems will gradually emerge in the coming years. In future work, technical personnel and researchers need to cooperate and incorporate the blockchain into the design of the medical framework. In this review, we demonstrated all of the potential scenarios of blockchain technology for patients and health care providers, offering large samples for further research. This only represents the beginning of the blockchain, and its development is expected to be more of a marathon than a sprint.

## Abbreviations

AI: artificial intelligence
CT: computed tomography
ECG: electrocardiogram
EHR: electronic health record
EMR: electronic medical record
IoT: Internet of Things
IPFS: interplanetary file system
PRISMA: Preferred Reporting Items for Systematic Reviews and Meta-analyses
